# WISP1 and Macrophage Migration Inhibitory Factor in Respiratory Inflammation: Novel Insights and Therapeutic Potentials for Asthma and COPD

**DOI:** 10.3390/ijms251810049

**Published:** 2024-09-18

**Authors:** Maria-Elpida Christopoulou, Alexios J. Aletras, Eleni Papakonstantinou, Daiana Stolz, Spyros S. Skandalis

**Affiliations:** 1 Laboratory of Biochemistry, Department of Chemistry, University of Patras, 26504 Patras, Greece; aletras@chemistry.upatras.gr; 2 Clinic of Pneumology, Medical Center-University of Freiburg, Faculty of Medicine, University of Freiburg, 79106 Freiburg, Germany; eleni.papakonstantinou@uniklinik-freiburg.de (E.P.); daiana.stolz@uniklink-freiburg.de (D.S.)

**Keywords:** WISP1, MIF, integrin, asthma, COPD, inflammation, extracellular matrix

## Abstract

Recent advancements highlight the intricate interplay between the extracellular matrix (ECM) and immune responses, notably in respiratory diseases such as asthma and Chronic Obstructive Pulmonary Disease (COPD). The ECM, a dynamic structural framework within tissues, orches-trates a plethora of cellular processes, including immune cell behavior and tissue repair mecha-nisms. WNT1-inducible-signaling pathway protein 1 (WISP1), a key ECM regulator, controls immune cell behavior, cytokine production, and tissue repair by modulating integrins, PI3K, Akt, β-catenin, and mTOR signaling pathways. WISP1 also induces macrophage migration inhibitory factor (MIF) expression via Src kinases and epidermal growth factor receptor (EGFR) activation. MIF, through its wide range of activities, enhances inflammation and tissue restructuring. Rec-ognized for its versatile roles in regulating the immune system, MIF interacts with multiple immune components, such as the NLRP3 inflammasome, thereby sustaining inflammatory pro-cesses. The WISP1–MIF axis potentially unveils complex molecular mechanisms governing im-mune responses and inflammation. Understanding the intricate roles of WISP1 and MIF in the pathogenesis of chronic respiratory diseases such as asthma and COPD could lead to the identi-fication of novel targets for therapeutic intervention to alleviate disease severity and enhance patient outcomes.

## 1. Introduction

Asthma is a chronic inflammatory condition that affects the airways, characterized by bronchial hyperresponsiveness, airway remodeling, narrowing of the lumen, and excessive mucus production [1]. Asthma is primarily a Th2-dominant immune response linked to type I hypersensitivity. Th2 cells secrete cytokines such as IL-4, IL-5, and IL-13, promoting IgE production and recruitment of eosinophils and mast cells [2]. Eosinophils and mast cells release inflammatory mediators that damage tissues and increase bronchial hyperresponsiveness, while dendritic cells (DCs) present allergens to T cells, initiating the Th2 response and priming of lymphoid cells [3]. Cytokines such as IL-33 and IL-25 released by epithelial cells further enhance inflammation and immune cell recruitment, perpetuating the cycle of asthma symptoms [1]. Notably, WNT signaling and downstream targets have been reported to be involved in the above events [4]. The activation of WNT/β-catenin signaling generally suppresses adaptive immune responses, inhibits the trafficking of inflammatory cells into the alveolar space, reduces the proliferation of activated Th2 cells, and decreases Th2 cytokine expression. Conversely, negative regulators of WNT signaling can reverse this inhibition. The suppression of β-catenin signaling attenuates airway remodeling, including smooth muscle growth and extracellular matrix (ECM) protein synthesis. While the effects of β-catenin-independent WNT signaling are less well-described, they include the modulation of airway smooth muscle contraction and the activation of inflammatory responses. Additionally, cross-talk between β-catenin-independent WNT signaling and pathways such as TGF-β signaling collectively drives airway remodeling [5].

Chronic Obstructive Pulmonary Disease (COPD) is a chronic, incurable lung disease characterized by sustained airflow limitation. It arises from a complex interaction between genetic factors and environmental exposures, with cigarette smoking being the primary risk factor [6]. Other contributing factors include age [7], gender, lung growth and development [8,9,10], exposure to harmful particles [11], socioeconomic status [12,13,14], asthma [15,16], airway hyperreactivity, chronic bronchitis [13], and infections [16]. The pathogenesis of COPD involves chronic airway inflammation, airway remodeling, and loss of alveolar parenchyma. Oxidative stress plays a crucial role, leading to lung cell injury, mucus hypersecretion, and enhanced inflammation via the production of reactive oxygen species (ROS) [17,18]. This stress disrupts the protease/anti-protease balance, resulting in tissue destruction and emphysema [19,20]. Increased synthesis and secretion of cytokines and chemokines (such as tumor necrosis factor-alpha (TNF-α), interleukin-1β (IL-1β), IL-6, and IL-8) perpetuate inflammation, while cell adhesion molecules (such as β2 integrins and ICAM-1) aid in neutrophil migration to lung tissue [21,22]. Additionally, goblet cell hyperplasia, a pathological change in response to harmful stimuli, occurs as part of the pathophysiology of COPD. This, along with increased mucoprotein (MUC) synthesis and mucus secretion, contributes to airway obstruction [23,24]. Alterations in WNT/β-catenin signaling, driven by cigarette smoke and fibroblast-derived WNT-5A, further impair epithelial proliferation and alveolar repair [25,26]. In addition, growth factors such as EGF and TGF-β contribute to airway remodeling and fibrosis during COPD [27,28,29]. In airway smooth muscle cells, cyclic adenosine monophosphate (cAMP), through protein kinase A (PKA) and/or the exchange proteins directly activated by cAMP (EPAC), inhibit inflammatory pathways activated by cigarette smoke extracts (CSEs) by blocking NF-κB and ERK activation and releasing the neutrophil chemokine IL-8. Furthermore, increased endothelin-1 (ET-1) levels in COPD patients, especially during exacerbations, contribute to pulmonary hypertension by promoting vasoconstriction and vascular remodeling [30,31,32]. ET-1 stimulates the production of C-reactive protein (CRP) and interleukin-6 (IL-6), which further amplify inflammation [33,34] while inducing the synthesis of vascular endothelial growth factor (VEGF), contributing to pathological changes in the vasculature [35].

Understanding the pathophysiology of respiratory diseases such as asthma and COPD is crucial, as these conditions, affecting millions of people worldwide, involve complex immune responses and airway changes. In asthma, immune dysregulation leads to airway inflammation and remodeling, making it essential to develop treatments that address both issues for better long-term outcomes. COPD shares similar mechanisms with asthma, but its heterogeneity complicates treatment. Current treatments often fall short, underscoring the need for personalized approaches. While biological therapies show promise, their high costs limit access. Therefore, understanding these diseases at the molecular level is vital for developing diagnostic tools and personalized treatments, as well as identifying new therapeutic targets to manage both inflammation and airway remodeling more effectively.

Recent advances in immunology have revealed a complex network of regulatory pathways that govern immune responses and inflammation [36]. One of the emerging areas of focus is the interaction between the immune system and the ECM [37]. The ECM, a structural and functional framework within tissues, plays a critical role in modulating immune responses [36,38,39]. The composition of the pulmonary ECM had proven difficult to elucidate until recent progress in mass spectrometry and quantification algorithms [40,41]. These technological advances have allowed researchers to elucidate the complex interactions between the ECM components and immune cells, revealing how changes in the ECM can affect inflammation and immunity. Understanding these ECM–immunity interactions is crucial for the development of novel therapeutic strategies for inflammatory and autoimmune diseases. Recent studies have identified WISP1 and Macrophage Migration Inhibitory Factor (MIF) as two candidate molecules implicated in the pathophysiology of lung diseases. WISP1 matricellular protein, a target gene of the WNT signaling, has been shown to modulate immune cell behavior and ECM remodeling in various pulmonary conditions [3,4,42,43]. On the other hand, MIF plays a pivotal role in regulating inflammation and immune responses within the lung microenvironment [44,45]. These molecules highlight the intricate interplay between the ECM components and immune pathways, suggesting their potential targeting for therapeutic interventions aimed at mitigating lung inflammation and restoring tissue homeostasis.

## 2. Background

### 2.1. WISP1

#### 2.1.1. WISP1 Structure, Expression, and Regulation

**Figure 1:**
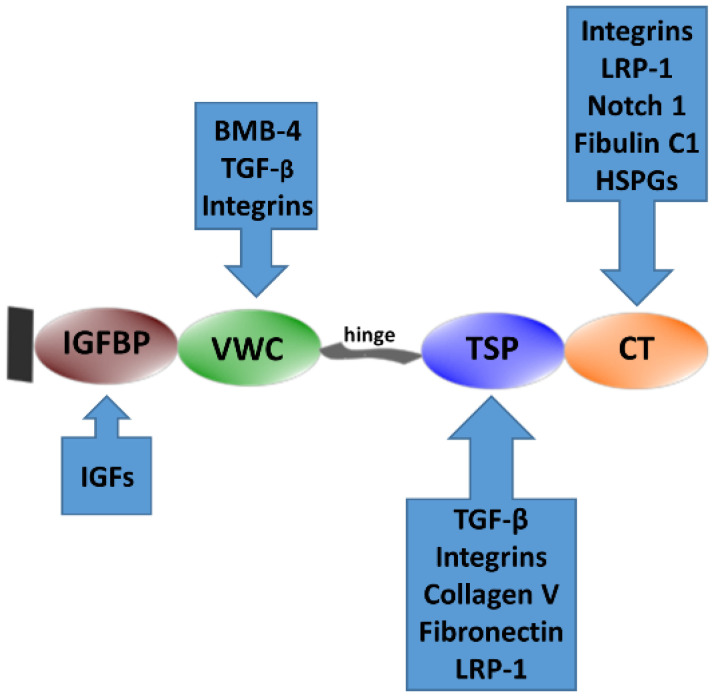
**Structure of CCN proteins.** A classical CCN protein features an N-terminal signaling peptide and four functional domains: (1) insulin-like growth factor-binding protein (IGFBP) domain, (2) von Willebrand factor type C (VWC) domain, (3) thrombospondin type 1 repeat (TSP-1) domain, and (4) cysteine knot carboxyl-terminal repeat (CT) domain. Each of these domains mediates interactions with various binding partners. For example, insulin-like growth factors (IGFs) bind to the IGFBP domain, while bone morphogenic protein 4 (BMP4) and transforming growth factor β (TGF-β) interact with the VWC domain. The TSP-1 domain interacts with heparin-sulfated proteoglycans (HSPGs), and the CT domain engages with LDL receptor protein 1 (LRP-1). Additionally, CCN proteins bind to a variety of cell surface receptors, including integrins, HSPGs, LRPs, and growth factors such as TGF-β, the vascular endothelial growth factor (VEGF), and BMP-4.

WISP1, also known as CCN4, is a member of the cellular communication network (CCN) family of growth factors [46,47], originally named from its first members: cysteine-rich angiogenic protein 61 (CYR61/CCN1), connective tissue growth factor (CTGF/CCN2), and nephroblastoma overexpressed protein (NOV/CCN3) [48]. All CCN proteins, except WISP2/CCN5, have an N-terminal signal peptide and four conserved domains: a region homologous to insulin-like growth factor-binding protein (IGFBP), von Willebrand factor type C (VWC), thrombospondin type 1 repeat (TSP-1), and cysteine knot (CT) [49]. These domains mediate interactions with extracellular proteins and cell surface receptors (Figure 1) [50,51,52]. Changes in the structure of CCN proteins, including the loss of specific domains, are linked to alterations in function, potentially contributing to disease development [46,51]. WISP1, in its complete form, comprises four distinct domains [53]. Research has shown a link between the absence of the VWC domain in WISP1 and the occurrence of invasive gastric carcinoma and cholangiocarcinoma [54,55,56]. The variant of WISP1 lacking the VWC domain is denoted as WISP1v. The complete WISP1 protein has 367 amino acids, with an estimated molecular mass of 40 kDa. It has 38 conserved cysteine residues and four possible glycosylation sites [53,57,58]. Studies have revealed that WISP1 is glycosylated, showing distinct patterns in different types of cancer cells and non-cancerous fibroblasts [59].

WISP1 is present in multiple tissues, including the epithelium, heart, kidney, lung, pancreas, placenta, ovaries, small intestine, spleen, and brain. Initially, WISP1 was identified as a target of the Wnt pathway in the mammary epithelial cell line C57MG, with several subsequent studies supporting its activation by β-catenin [57,60,61,62]. There are 19 genes encoding WNT proteins in humans, including WNT1, WNT2, WNT2B, WNT3, WNT3A, WNT4, WNT5A, WNT5B, WNT6, WNT7A, WNT7B, WNT8A, WNT8B, WNT9A, WNT9B, WNT10A, WNT10B, WNT11, and WNT16 [63,64]. They are secreted glycoproteins that act as signaling molecules by binding to a complex of heterodimeric receptors, which consist of a receptor from the Frizzled (Fz) family and a co-receptor such as the Low-Density Lipoprotein Receptor-Related Protein (LRP) 5/6, Ryk, or ROR [65].

In brief, Wnt signaling can be activated through either the canonical or non-canonical pathways, depending on the receptors, co-receptors, and intracellular regulators involved [63]. The canonical Wnt/β-catenin pathway involves the activation of β-catenin, which occurs when the Wnt receptor undergoes a conformational change and recruits Axin [66]. The Fz receptor interacts with Disheveled (Dsh), promoting its interaction with Axin. The LRP co-receptor also binds Axin in a Wnt-dependent manner, with phosphorylation by GSK-3β and CK1 kinases regulating this interaction [67]. In the absence of Wnt, β-catenin is kept at low levels by a destruction complex (APC/GSK-3β/CK1/Axin), where it is phosphorylated, ubiquitinated, and degraded [68]. When Wnt is present, Axin dissociates from the destruction complex, preventing β-catenin phosphorylation and degradation, allowing β-catenin to accumulate, enter the nucleus, and activate transcription factors such as the T-cell factor/lymphoid enhancer-binding factor (TCF/LEF), which regulate developmental processes [69].

Non-canonical Wnt signaling pathways, which are independent of β-catenin, are classified into Wnt/JNK and Wnt/Ca^2+^ pathways based on their main intracellular mediators [70]. Unlike canonical pathways, non-canonical pathways do not involve the co-receptor LRP5/6 [71]. The Wnt/JNK pathway activates protein kinases such as JNK and Rho kinase [72]. This pathway regulates gene expression, cell polarity, and cytoskeletal reorganization in the construction of other proteins [72]. In the Wnt/Ca^2+^ pathway, Wnt proteins bind to Fz receptors, activating heterotrimeric G proteins that interact with these receptors. This activation triggers phospholipase C-β (PLC-β) to hydrolyze PIP2 into IP3 and DAG, leading to increased intracellular Ca^2+^ levels and PKC activation [73]. The rise in Ca^2+^ activates calmodulin, which then activates calcineurin phosphatase. Calcineurin dephosphorylates the transcription factor NFAT, regulating gene transcription involved in cell fate and migration [74].

In humans, it was first detected in cells with elevated levels of WNT-1 protein. However, it was further revealed that WNT3A also induces WISP1 expression, indicating that WISP1 is a target gene of the canonical WNT/β-catenin pathway [61,75,76]. Furthermore, research using animal models of asthma revealed that although the overall β-catenin levels in the airway smooth muscle layer remained constant, its activity was heightened. This was demonstrated by the increased expression of genes such as *Axin2*, *Wisp1*, and *c-Myc* [77]. Interestingly, there was an increase in WNT-5A, which is highly associated with asthma with high Th2 levels, suggesting a possible role of the non-canonical pathway in this case [76,78]. Given the involvement of WNT-5A in non-canonical WNT signaling and its association with conditions such as asthma, lung cancer, and COPD, it is plausible that WNT-5A might influence β-catenin activity indirectly [25,79,80,81,82]. Also, in ventilator-induced lung injury (VILI), initial attention was directed towards the canonical WNT/β-catenin signaling pathway and its association with WISP1 [83,84]. Further studies revealed the activation of both canonical and non-canonical WNT signaling pathways [79,85]. Intriguingly, while activation of non-canonical signaling (WNT-5A) was associated with VILI promotion, it also led to the upregulation of WISP1 expression [50].

The promoter region of *Wisp-1* contains binding sites for several transcription factors, including the cAMP response element (CRE) and transcription factor TCF/LEF [75,86]. When canonical WNT signaling is activated, β-catenin accumulates in the cytoplasm and translocates to the nucleus, where it binds to TCF/LEF transcription factors, initiating the expression of WNT target genes, including WISP1 [87]. This regulation is modulated by dynamic protein modifications, such as phosphorylation, which influence the activity of the β-catenin degradation complex [68]. Additionally, within the nucleus, the function of β-catenin is further modulated by its interaction with transcriptional coactivators such as the CREB binding protein (CBP) and p300 [88]. WNT-3A primarily triggers the β-catenin/TCF pathway, while WNT-5A induces nuclear translocation of β-catenin without activating β-catenin/TCF [81]. Instead, the action of WNT-5A relies on CREB activation and the subsequent CREB-dependent regulation of WISP1 [89,90]. However, in certain disease contexts, such as atherosclerosis or aging-related conditions, impaired phosphorylation of CREB by WNT-5A abolishes the CREB-dependent regulation of WISP1, negatively impacting cell survival [90]. Furthermore, WNT-5A can activate β-catenin-dependent transcription depending on the time and the presence of specific receptors, such as Frizzled Class Receptor 4 (FZD4) and LRP5 [81,91,92,93], adding complexity to WISP1 regulation by different Wnt ligands. Therefore, WISP1 expression is intricately controlled by the coordinated action of β-catenin, TCF/LEF, and CREB, with the CREB binding site on the WISP1 promoter playing a crucial role in β-catenin-dependent transcriptional activation [62,94,95]. Therefore, further confirmatory studies are required to determine which specific WNT signaling pathway(s) is responsible for WISP1 production in the lungs. Existing studies have provided information on the involvement of both canonical and non-canonical WNT pathways in the regulation of WISP1 expression. However, further investigations are needed to elucidate the precise mechanisms underlying this relationship.

WISP1 expression can also be influenced by various extracellular signals, including profibrotic growth factors, cytokines, and mechanical stretching. Furthermore, it appears that WISP1 is upregulated in lung tissues following hepatic ischemia–reperfusion injury (IRI) [96]. Studies have shown that the expression of IL-1β and TNF-α precedes WISP1 expression in vivo in cardiomyocytes and can stimulate WISP1 expression in neonatal rat ventricular myocytes in vitro [97,98]. In cardiomyocytes, TNF-α-induced expression of WISP1 involves ERK1/2 activation and CREB signaling [98]. Additionally, in human prostate fibroblasts (HPrF cells), TNF-α induces the expression and secretion of WISP1 variants, WISP1v1 and WISP1v2 [99]. However, WISP1 is also a downstream gene of the TGF-β signaling pathway in primary lung fibroblasts and hepatic fibrosis, suggesting that its regulatory mechanisms can vary in a cell-type or cell-context manner [100,101]. Moreover, the toll-like receptor 4 (TLR4) plays a significant role in regulating WISP1 expression in macrophages during ventilator-induced lung injury (VILI), and the ability of WISP1 to promote cytokine secretion depends on the TLR4 presence [101]. Mechanical stretching enhances WISP1 expression in lung fibroblasts, potentially contributing to their hypertrophy via the Hedgehog pathway [102]. Further, in alveolar type 2 (ATII) cells, mechanical stretching upregulates WISP1 in a hyaluronan- and MyD88-dependent manner, and blocking WISP1 prevents an epithelial–mesenchymal transition (EMT) [61,103].

#### 2.1.2. Roles of WISP1 in Signaling Pathways and Cellular Processes

WISP1 plays a critical role in cell differentiation, proliferation, tissue regeneration, and the regulation of signal transduction [96,104,105,106,107,108]. CCNs, including WISP1, do not bind to specific membrane receptors but interact with multiligand receptors, primarily integrins such as α5β1, αvβ3, and αvβ5, to regulate intracellular signaling [49,104,109]. This interaction triggers a cascade of signaling pathways, including FAK, Src kinases, EGFR, RAS/RAF/MEK/ERK, NF-κB, TGF-β, and PI3K/Akt, depending on the cellular context [104,109,110,111]. Functionally, WISP1-activated signals regulate both beneficial processes, such as tissue repair, and potentially harmful processes, such as chronic inflammation and cancer progression [47,83,104,112,113,114,115,116,117,118].

WISP1, through the αvβ3 integrin, enhances TLR4-mediated ERK signaling and increases the release of TNF-α in macrophages, thereby contributing to inflammation in sepsis-induced lung injury [83]. Moreover, WISP1 has been reported to further increase LPS-induced cytokine release, which is dependent on both TLR4 and the integrin β5. Specifically, WISP1, through its interaction with the integrin αvβ5, affects macrophages by enhancing TLR4-mediated inflammatory signaling via the ERK, JNK, and p38 pathways, leading to an increased cytokine release [113]. It also promotes adhesion in the lung epithelial cells, potentially exacerbating local inflammation. WISP1 induced the adhesion in A549 epithelial cells mainly through its interaction with the αvβ3 and β1 integrins, mediated by the C-terminal domain of the WISP1 protein [47]. Pretreatment of A549 cells with neutralizing antibodies targeting the αvβ5 and β1 integrins partially inhibited adhesion induced by the full-length WISP1. The inhibitory effects were more pronounced with the combined use of antibodies against both the αvβ5 and β1 integrins, highlighting their important role in mediating WISP1-induced adhesion. The integrin αvβ3 also contributed to this process, although its specific role relative to αvβ5 and β1 was comparatively less pronounced, suggesting the involvement of multiple integrins in the WISP1-mediated adhesion process in A549 cells. These actions collectively contribute to the pathogenesis of Acute Lung Injury (ALI) in the setting of sepsis and mechanical ventilation. Moreover, WISP1 interacts with the integrins α5 and αv to significantly influence chondrocyte behavior. In combination with TGF-β1, WISP1 was shown to promote chondrocyte de-differentiation [114]. Specifically, chondrocytes exposed to WISP1 and TGF-β1 showed reduced levels of chondrogenic markers such as collagen type II and SOX9, along with an increased expression of de-differentiation markers such as collagen type I and Runx2. Silencing the integrin α5 worsened WISP1-induced de-differentiation, reversed by its upregulation, whereas upregulating the integrin αv exacerbated the effect. Moreover, WISP1 facilitated wound healing by promoting dermal fibroblast migration and proliferation through the α5β1 integrin-mediated activation of ERK/JNK signaling pathways [115]. WISP1 also regulated the inflammatory response mediated by TNF-α. Specifically, WISP1, via the integrin α5β1, counteracted TNF-α‘s inhibitory effect on collagen (COL1A1) and fibronectin (FN) mRNA expression in dermal fibroblasts, potentially by modulating the downstream NF-κB/p65 signaling pathway [115]. In another line of research, WISP1 in the context of non-alcoholic steatohepatitis (NASH)-associated chronic liver disease induced MRTF activation and enhanced myofibroblast gene expression related to cytoskeletal dynamics and motility via the integrins αVβ1/3/5/8 and α11β1 [116]. This involved downstream Rho activation and actin polymerization, contributing to fibrosis progression [116,117,118]. Another interesting aspect of WISP1–integrin interaction is its dual role in glioblastoma (GBM) progression. WISP1, secreted by glioma stem cells (GSCs), plays a pivotal dual role in GBM by signaling through the integrin α6β1. Within GSCs, WISP1 acts in an autocrine manner, binding to the integrin α6β1 to activate the Akt pathway, thereby sustaining GSC proliferation and tumor sphere formation critical for tumor growth. Simultaneously, WISP1 functions in a paracrine fashion on tumor-associated macrophages (TAMs), particularly the M2 phenotype, promoting their survival through integrin α6β1-mediated Akt activation [119].

In addition to integrins, CCN proteins interact with a variety of cell surface receptors, such as heparan sulfate proteoglycans (HSPGs), proteins associated with LRPs, and the cation-independent mannose 6-phosphate receptor (M6P/IGF-2R), which may also function as co-receptors or accessory receptors for other receptor systems [83,104,109]. Moreover, they interact with growth factors such as TGF-β, VEGF, and bone morphogenetic protein (BMP-4) [49,120,121]. The N- and C-termini of CCN proteins are connected by a flexible ‘hinge’ region, which is susceptible to proteolysis. This can lead to the formation of truncated CCN proteins exhibiting both shared and unique biological activities compared to full-length proteins [51,54].

**Figure 2:**
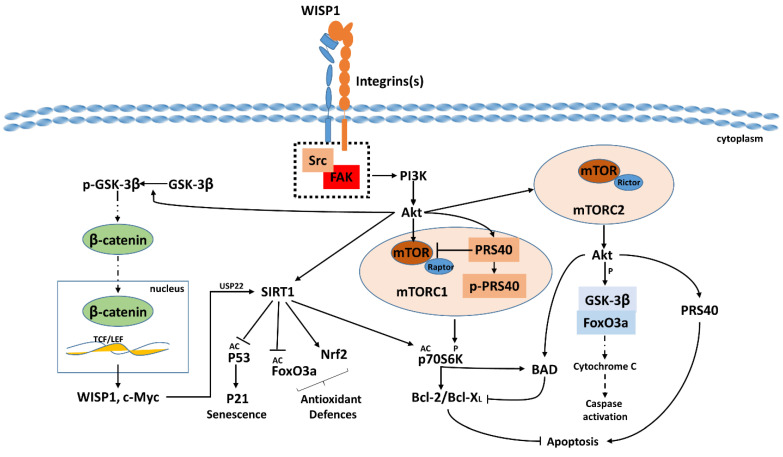
**WISP1 in Cellular Stress Responses.** WISP1, as a downstream target of WNT signaling, plays a crucial role in cytoprotection and cell survival through various molecular pathways. WISP1 binds integrins and activates the PI3K/Akt pathway, inhibiting GSK-3β and stabilizing β-catenin, which enhances the transcription of anti-apoptotic genes. WISP1 modulates apoptotic pathways by influencing mitochondrial signaling and preventing cytochrome c release, thereby inhibiting caspase activation. WISP1 also upregulates Bcl-XL, which sequesters pro-apoptotic proteins. Additionally, WISP1 mitigates p53-mediated apoptosis through PI3K/Akt activation, preventing apoptosome formation. It establishes a positive feedback loop by inducing its own expression and enhances SIRT1 activity, which modulates PI3K/Akt signaling. WISP1 suppresses FoxO3a activity through PI3K/Akt-mediated phosphorylation and interacts with the mTOR pathway, activating mTORC1 to promote cell growth and proliferation. Collectively, these mechanisms underscore the role of WISP1 in cell survival, tissue repair, and tumorigenicity; AC (acetylation); and p (phosphorylation).

WISP1, as a downstream target of Wnt signaling, plays a crucial cytoprotective role against oxidative stress and cell injury across various tissues through multiple molecular pathways (Figure 2). It enhances bone formation and repair, impacts tumorigenesis, and supports cellular hypertrophy [122,123,124,125,126]. Notably, WISP1 improves cell survival during cardiomyocyte injury, prevents vascular cell death, and supports neuronal survival under oxidative stress, while in cases of lung injury, it mitigates inflammation and promotes tissue healing [53,61,104,126,127,128,129,130,131,132]. Mechanistically, WISP1 activates the PI3K/Akt pathway, which is essential for cell growth and survival. This activation leads to the inhibition of GSK-3β, preventing β-catenin degradation, allowing β-catenin to translocate to the nucleus and initiate transcription of anti-apoptotic genes [133]. In this way, WISP1 directly influences apoptotic mitochondrial signaling pathways. It inhibits the activity of pro-apoptotic proteins such as Bad, Bax, and Bim, which normally promote apoptosis by facilitating mitochondrial membrane permeabilization and cytochrome c release. By blocking these proteins, WISP1 prevents cytochrome c release, thereby inhibiting caspase activation and ultimately preventing apoptosis [111,127]. Furthermore, WISP1 has been shown to mitigate p53-mediated apoptosis following DNA damage by activating the PI3K and Akt pathways. Activation of Akt by WISP1 further inhibits the cytochrome c release from mitochondria, preventing the formation of apoptosomes and subsequent caspase activation [102]. Additionally, WISP1 upregulates the expression of anti-apoptotic protein Bcl-XL, which sequesters pro-apoptotic Bax and antagonizes BH3-only proteins such as PUMA and NORA, crucial for inducing cytochrome c release [133,134]. This dual action of WISP1 not only suppresses apoptotic signaling downstream of p53 but also enhances cell survival mechanisms, including the regulation of autophagy, thereby promoting cellular tumorigenicity [102,128,135]. Notably, WISP1 exhibits autoregulatory mechanisms by inducing its own expression, establishing a positive feedback loop that sustains its cytoprotective effects over time [53,128,136].

Further, WISP1 enhances the activity of Sirtuin 1 (SIRT1), a protein crucial for cell regulation and longevity, through an autoregulatory mechanism. WISP1 can enhance SIRT1 activity, which in turn can modulate the PI3K/Akt pathway by deacetylating key regulatory proteins. This modulation can create a feedback loop where SIRT1 activity helps maintain optimal PI3K/Akt signaling [137,138]. Concurrently, WISP1 suppresses the activity of the mammalian forkhead transcription factor FoxO3a [139,140]. The PI3K/Akt pathway can phosphorylate FoxO3a, leading to its inactivation and nuclear exclusion, thereby reducing its transcriptional activity. WISP1’s suppression of FoxO3a activity may be partly mediated through the activation of the PI3K/Akt pathway. At the same time, SIRT1 deacetylates and activates FoxO3a, suggesting a complex interplay where WISP1’s effects on SIRT1 and Akt balance the activity of FoxO3a.

WISP1 targets several components of the mechanistic target of the rapamycin (mTOR) pathway, which regulates cell survival and proliferation [138]. WISP1 activates the PI3K/Akt pathway upstream of mTOR, leading to the Akt-mediated inhibition of TSC2, which in turn activates mTORC1. This activation promotes the phosphorylation of proteins such as p70S6K and 4EBP1 through the regulation of the 40 kDa proline-rich Akt substrate (PRAS40), thereby enhancing cell growth and proliferation. Additionally, mTORC1 upregulates anti-apoptotic proteins and downregulates pro-apoptotic factors, facilitating cell survival while also influencing upstream mTOR pathways, including interactions with protein kinase B/Akt [112,126,136]. Through these actions, WISP1 enhances cell growth and proliferation by promoting protein synthesis and cell cycle progression while also supporting cell survival by upregulating anti-apoptotic proteins and inhibiting pro-apoptotic factors.

In the context of lung diseases, WISP1 might play a protective role in lung tissue under normal physiological conditions or in response to injury by aiding in tissue repair and regeneration [106,126,141,142]. However, the promotion of cellular survival pathways such as PI3K/Akt and mTOR by WISP1 can lead to fibrosis and cancer if its activity becomes unchecked (Table 1). In diseases such as idiopathic pulmonary fibrosis (IPF), WISP1’s enhancement of cell survival and proliferation can exacerbate fibrosis by encouraging the persistence and growth of fibrogenic cells [61,143]. In lung cancer, the pathways activated by WISP1 can contribute to tumorigenesis by promoting unchecked cellular proliferation and survival [138,144]. Additionally, WISP1 may modulate inflammatory responses, which, while beneficial for repair, can lead to tissue damage in chronic conditions such as COPD or asthma if excessively activated [43]. Furthermore, the ability of WISP1 to regulate its own expression through feedback mechanisms can sustain beneficial effects but may also perpetuate harmful ones if overactivated, potentially contributing to disease progression [128]. Thus, while WISP1 has protective and supportive roles in lung tissue repair and regeneration, its overactivation can result in pathological conditions such as fibrosis, cancer, and chronic inflammation, highlighting the need to balance its activity for therapeutic purposes [43].

### 2.2. MIF

MIF is a multifunctional cytokine involved in various physiological and pathological processes [45,75,151,152,153]. MIF is known for its distinct enzymatic properties, specifically displaying phenylpyruvate tautomerase and D-dopachrome tautomerase (DDT) activities [154,155]. It is produced by a variety of cells, including immune cells such as macrophages, B cells, and T cells, as well as non-immune cells such as endocrine, endothelial, and epithelial cells, and fibroblasts [44,104,156,157]. MIF is primarily found in the cytosol, but it is also present in the cell membrane, likely bound to a receptor. MIF stands out among cytokines due to its continuous production within cells and storage in intracellular pools, enabling rapid release without requiring new protein synthesis [158]. Additionally, it possesses the ability to amplify its own expression [104,159,160]. Unlike most secreted proteins that possess an N-terminal signaling peptide directing their transport through the endoplasmic reticulum and Golgi apparatus, MIF lacks this signaling sequence [158,161,162]. This absence suggests that MIF utilizes an unconventional secretion pathway involving factors such as the transport protein p115 (also known as USO-1) and possibly an ATP-binding cassette transporter such as ABCA1 [163,164]. Recent studies have also highlighted MIF’s presence in exosomes released from adenocarcinoma cells, proposing a novel mechanism for MIF’s extracellular interactions [165,166]. However, the occurrence of MIF in exosomes from other cell types remains to be confirmed.

#### 2.2.1. Roles of MIF in Signaling Pathways and Cellular Processes

MIF exerts its biological activity dependent on tissue and physiological or disease context primarily by interacting with the cell surface receptor CD74 [167]. This interaction recruits CD44 to form a receptor complex and phosphorylation of the intracellular domains of both proteins to initiate signal transduction [104,168,169]. The intracellular domain of CD44 binds to the SH2–SH3 domains of Src kinases, inducing a conformational change that exposes their active sites and simultaneously triggers a rapid but transient autophosphorylation at the Y416 residue of Src [168,170]. Subsequently, Src kinases initiate various downstream signaling pathways, including the mitogen-activated protein kinase (MAPK), PI3K/Akt, NF-κB, and ERK1/2-MAPK pathway, resulting in the activation of cyclin D1 and the transcription factors ETs and AP-1 [171]. Additionally, the MIF-mediated activation of Src kinases triggers the PI3K/Akt pathway, promoting cell proliferation and gene expression while inhibiting p53-dependent apoptosis. Akt activation leads to the phosphorylation and activation of the E3 ligase Mdm2, facilitating the degradation of p53 and thereby inhibiting p53-dependent gene transcription. Moreover, MIF directly suppresses p53 function through the sequential activation of the ERK1/2, PLA2, COX-2, and PGE2 pathways, contributing to its role in apoptosis inhibition and proliferation promotion [172].

Alternatively, upon binding to the CD74/CD44 receptor complex, MIF stimulates toll-like receptor 4 (TLR4) expression and induces the release of the intracellular domain of CD74 (CD74-ICD) through proteolytic cleavage and endocytosis [170,173]. This proteolysis is mediated by the Signal Peptide Peptidase-like 2a (SPPL2a) protease present in lysosomes and late endosomes [174,175]. Once released, the CD74-ICD translocates to the nucleus, where it interacts with transcription factors such as NF-κB (specifically the p65/RelA subunit) and the coactivator TAFII105, thereby enhancing NF-κB activation. It also interacts with RUNX1-3, further regulating their activity [176]. Moreover, the proteolytic shedding of CD44 can also occur under certain conditions [177]. Therefore, the possibility of the interaction and/or coordinated actions of both the intracellular domains of CD74 and CD44 within the nucleus should not be excluded and warrants further investigation.

The activation of NF-κB leads to the induction of the NLRP3 protein, facilitating the assembly of the inflammasome [178]. This complex involves interactions between ASC, caspase-1, vimentin, and the NOD-, LRR-, and pyrin domain-containing protein 3 (NLRP3), which are crucial for the maturation of pro-IL-1β and pro-IL-18 into their active forms, resulting in an increased production of IL-1β and IL-18 [179]. MIF plays a crucial role in NLRP3 inflammasome activation, interacting directly with NLRP3 and vimentin [178]. It is implicated both intracellularly and extracellularly, affecting the IL-1β release in response to human monocytes to RNA-containing U1 small nuclear ribonucleoprotein (snRNP) immune complexes [180]. Additionally, U1 snRNP immune complexes upregulate the expression of the MIF receptors CD74 and CD44, suggesting that MIF can modulate NLRP3-dependent IL-1β release through interactions with these receptors, both inside and outside the cell [181].

Moreover, MIF has been reported to regulate NF-κB activation by engaging directly with the Thioredoxin-Interacting Protein (TXNIP), a recognized inhibitor of the NF-κB function [182]. The inhibition of TXNIP leads to an increase in the production of inflammatory proteins such as pro-IL-1β, pro-IL-18, and NLRP3 [181]. This process is believed to disrupt the association between TXNIP and regulatory molecules such as histone deacetylases (HDACs) and p65 [182]. As a result, MIF promotes NF-κB-mediated gene expression, leading to the upregulation of genes associated with inflammation, cell survival, and other cellular processes regulated by NF-κB [182].

In this context, MIF also regulates the expression of TLR4, which allows frontline defense cells, such as macrophages, to rapidly produce pro-inflammatory cytokines [44,183]. Thus, the overexpression of TLR4 and the regulation of cell survival are key mechanisms by which MIF promotes pro-inflammatory responses. The intricate interplay between CD74-ICD, NF-κB, and other transcription factors underscores MIF’s pivotal role in regulating inflammatory processes and innate immune responses [44,184].

MIF can also be endocytosed and interact with intracellular proteins such as the JUN activation domain-binding protein 1 (JAB1/CSN5) or the ribosomal protein S19 to modulate its intracellular signaling [185,186]. The interaction between MIF and JAB1, mediated by the CXXC motif of MIF, suppresses the activity of JAB1-induced c-Jun N-terminal kinase (JNK) and activates AP-1 [186,187]. Further, MIF functions similarly to chemokines by promoting cell migration, particularly in inflammatory and atherogenic leukocyte recruitment, through interactions with the CXC chemokine receptors CXCR2 and CXCR4 [188,189,190]. MIF can bind to the G-protein-coupled receptors CXCR2, CXCR4, and CXCR7 and induce downstream activation of the MAPK pathway [188,189,191]. This binding can occur independently or in conjunction with CD74, leading to the formation of complexes that result in a calcium influx and rapid integrin activation [189].

#### 2.2.2. Involvement of MIF in Immune Responses and Inflammation

MIF plays a vital role in inflammatory processes and the regulation of innate immunity. This is highlighted by its unique ability among cytokines to counteract the anti-inflammatory effects of glucocorticoids [153,192,193]. Moreover, MIF has an important role in the defense of the innate immune system against acute viral and bacterial infections, tuberculosis, and protozoal diseases [194,195,196,197,198]. This cytokine is secreted in response to various extracellular stimuli, including microbial products such as LPS, pro-inflammatory cytokines such as TNF-α and interferon-gamma (INF-γ), and the activation of specific antigens, giving MIF characteristics of alarmins or DAMPs [44,104,194,199,200,201,202,203,204]. Upon its release into tissues and circulation, MIF acts as a pro-inflammatory cytokine with both autocrine and paracrine effects.

Initially identified as a soluble factor produced by activated lymphocytes, MIF inhibits macrophage migration in delayed-type hypersensitivity studies [205,206]. MIF promotes directly or indirectly the production or expression of several pro-inflammatory molecules, including cytokines and growth factors such as TNF-α, IFN-γ, IL-2, IL-6, IL-8, and the macrophage inflammatory protein 2 [207,208,209,210,211]. Meanwhile, recent findings suggest that MIF influences the release of IL-1α, IL-1β, and IL-18 not through direct effects on their transcription or translation but rather by activating the NLRP3 inflammasome [212]. However, MIF inhibitors showed no effect on NF-κB or LPS-induced IL-1β/NLRP3 production, suggesting complex regulatory pathways [213].

MIF also induces the production of nitric oxide (NO) [158,214], COX-2, and prostaglandin E2 (PGE2) [104,215,216]. Interestingly, recent studies suggest that many key actions of MIF extend beyond inflammation. Despite being typically characterized as a pro-inflammatory cytokine, MIF is constitutively expressed in most human tissues, often at high levels, which is atypical for classical pro-inflammatory cytokines. Moreover, MIF is highly expressed during embryonic development, and its levels decrease in adulthood, indicating a potential role as a growth factor. This dual functionality highlights the complex role of MIF in both physiological and pathological processes [45]. Furthermore, the pro-inflammatory properties of MIF are mediated, at least in part, by its ability to counteract the immunosuppressive effects of glucocorticoids [207,210]. It has also been reported that MIF attenuates the suppressive effect of dexamethasone on IL-6 production by nasal polyps [217].

## 3. Molecular Mechanisms Involving WISP/MIF in Asthma and COPD

### 3.1. WISP1 in Asthma

Asthma is a chronic airway disease with high prevalence in children as well as adults. The hallmark pathological features of asthma include eosinophilic airway inflammation and structural changes known as airway remodeling, contributing to irreversible lung function decline. Cells implicated in airway remodeling encompass T-lymphocytes, airway epithelial cells, airway smooth muscle (ASM) cells, and lung fibroblasts [35,36,37]. Yet, the intricate dysregulation of ASM cells plays a pivotal role in the pathophysiology of the disease, making it a central target for therapeutic interventions in respiratory diseases such as asthma.

WISP1 is a key factor in ASM hypertrophy and proliferation in asthma, crucial aspects of airway remodeling that contribute to symptoms such as airflow obstruction and airway hyperresponsiveness [132]. The pathogenic role of WISP1 in asthma was first identified in a study using a rat model of allergic asthma [218]. This study demonstrated that WISP1 expression was upregulated in the airway remodeling model, showing significantly higher levels in lung tissue than in controls. Neutralizing antibodies against WISP1 partially inhibited pathological remodeling. This study also revealed that WISP1 treatment significantly increased lung fibroblast proliferation via the Akt signaling pathway. WISP1 may play a critical role in airway remodeling by inducing abnormal activation of lung fibroblasts, affecting ECM deposition similar to what is seen in hepatic fibrosis, myocardial remodeling, and lung fibrosis. Depletion of WISP1 significantly inhibited OVA-induced collagen deposition [218]. WISP1 treatment led to a collagen release from lung fibroblasts and increased levels of the ECM components Col1alpha1 and FN1 through the Akt pathway-mediated phosphorylation of GSK-3β [218]. In vitro, it was further shown that WISP1 could induce hBSMC hypertrophy and proliferation, along with upregulation of levels of PI3K, p-Akt, and p-GSK-3β, similar to TGF-β [76]. Specifically, TGF-β activated WISP1 expression via PI3K-dependent Akt/GSK-3β signaling. The Akt inhibitor partially suppressed WISP1- and TGF-β-induced abnormal activation of hBSMCs, implicating WISP1 in non-canonical TGF-β signaling [76]. Moreover, WISP1 was shown to sustain its own expression through autoregulation, possibly enhancing ASM remodeling [76].

WISP1 expression is influenced by β-catenin, a key component of the WNT signaling pathway [57]. However, it has been reported that while there may not be significant differences in β-catenin levels in the ASM between asthmatic and non-asthmatic individuals, the expression of genes controlled by β-catenin, including WISP1, is increased in a mouse model of OVA-induced asthma [77]. Another study suggested that the WNT/β-catenin pathway influences airway remodeling in asthma by modulating the expression of key regulators such as c-Myc and cyclin D1, potentially through interactions with the p38 MAPK pathway [219]. Pharmacological inhibition of β-catenin/CBP interaction using the small molecule ICG-001 prevented smooth muscle remodeling and expression of ECM proteins in both in vitro and in vivo asthma models, while β-catenin signaling was necessary for TGF-β-induced expression of ECM proteins by human ASM cells and pulmonary fibroblasts [77].

In the context of asthma, TNF-α is known to be a significant inflammatory mediator that contributes to the pathogenesis of the disease (1). TNF-α promotes the progression of severe asthma that is characterized by Th1 airway inflammation, while it also enhances Th2 inflammation in mild/moderate allergic asthma (1). It has been reported that TNF-α induces a significant inflammatory response in human ASMCs, including the upregulation of chemokines and cytokines linked to asthma and the activation of the signaling pathways associated with inflammation and remodeling [220]. Specifically, TNF-α enhanced the release of proteins such as ET-1, DKK1, GM-CSF, WISP1, and MMPs, which regulate cell proliferation, differentiation, and apoptosis. Since WISP1 is a target of WNT/β-catenin signaling and TNF-α upregulated WISP1 expression, this study suggests a mechanistic link between the TNF-α and Wnt pathways in regulating WISP1, probably via MAP kinases and Akt/CREB signaling. These findings indicated that TNF-α-driven WISP1 expression plays a role in the pathological changes seen in asthma, highlighting ASMCs as potential therapeutic targets in early-stage inflammation-associated chronic lung diseases. In another study, it appears that WISP1 may play a role in the airway remodeling process in asthma, and its exacerbation was modulated by Mycobacterium vaccae (*M. vaccae*) inhalation [221]. *M. vaccae* treatment led to reduced levels of the inflammatory cytokines IL-5, IL-13, and TNF-α, as well as OVA-specific IgE. This study also found that *M. vaccae* inhalation downregulated the expression of WISP1 mRNA in the pulmonary tissue of asthmatic mice, suggesting that WISP1 downregulation might contribute to this anti-inflammatory environment by reducing signals that promote inflammation and tissue remodeling [221].

Polymorphisms in the WISP1 gene are also associated with lung function in asthma [218]. One of the earliest studies suggesting a genetic link between WNT signaling and asthma was conducted by Sharma et al. [222]. They demonstrated that WNT signaling genes are associated with impaired lung function in asthmatic children. Specifically, WISP1 was associated with FEV1 and FVC, while WNT inhibitory factor-1 (WIF-1) was linked to FVC and the FEV1/FEV ratio, but not with FEV1 alone [222]. This indicates that WISP1 has a role in lung function, which can be indirectly linked to structural changes and ECM deposition in the airways. Furthermore, an SNP in the WISP1 gene (rs2929973) was significantly associated with FEV1 in asthmatic children, suggesting that genetic variations in WISP1 can influence lung function and potentially ECM deposition. Furthermore, a genome-wide association study (GWAS)-based analysis prioritizing asthma-susceptible genes found an enrichment of cytokine–receptor interactions and WNT signaling pathways in asthma pathogenesis [223]. Additionally, this study identified a new susceptibility locus near WNT signaling genes, particularly SNPs near FZD3 and FZD6.

### 3.2. MIF in Asthma

Overall, MIF levels appear to be higher in patients with asthma than in healthy individuals and are positively associated with disease severity [224]. MIF expression was found to be higher in bronchoalveolar lavage of patients with asthma [225]. Treatment with phorbol myristate acetate (PMA) showed that activated eosinophils were an important source of MIF [226]. In another study, higher levels of MIF were detected in the serum and sputum of symptomatic patients than in asymptomatic patients, whereas control subjects showed lower levels of MIF [224]. Animal studies using models of allergic lung inflammation supported a role for MIF in asthma pathogenesis [227,228,229,230,231]. Furthermore, in an OVA-sensitized mice model, a deficiency or inhibition of MIF led to reduced lung inflammation and hyperresponsiveness, indicating that MIF could be a promising target for therapeutic interventions [225,227].

In asthma, MIF has been shown to be involved in airway remodeling by promoting autophagy in ASM cells through binding to the CD74 receptor [225]. Results from OVA-challenged mice also indicated that MIF knock-down mice showed reduced ASM production, collagen deposition, and TGF-β1 accumulation compared to wild-type (WT) mice, emphasizing the role of MIF in airway remodeling [225]. In another study, it was demonstrated that MIF promotes ASM cell proliferation via ERK1/2/Drp1 axis-mediated autophagy activation and subsequent E-cadherin reduction [232]. Moreover, the MIF-specific suicide substrate and irreversible inhibitor 4-IPP effectively alleviated airway hyperresponsiveness and remodeling in OVA-induced asthma models by repressing aberrant mitochondrial fission-mediated autophagy activation [232].

In vitro evidence has shown that MIF promotes the proliferation of ASM cells and the migration of eosinophils [233,234]. However, MIF inhibition should be further evaluated and personalized as a therapeutic option, considering that MIF’s association with asthma is particularly linked to Th2-related responses rather than classic Th1 inflammation [227,228,235]. This suggests a role for MIF in promoting lung allergic inflammation. Moreover, recent research has revealed that MIF induces glucocorticoid resistance in severe neutrophilic asthma by reducing the activity of annexin-A1, a glucocorticoid-regulated protein that typically inhibits neutrophil accumulation in inflamed areas [213]. Additionally, heightened NLRP3 inflammasome activation correlates with increased MIF levels [213]. Thus, targeting MIF may represent a promising therapeutic strategy for managing severe neutrophilic asthma by enhancing glucocorticoid responsiveness and mitigating inflammation.

In addition, the −173 G/C single-nucleotide polymorphism in the MIF promoter is noted to influence MIF gene expression, with higher expression associated with the C nucleotide [236,237,238]. Studies have found a higher frequency of the MIF −173CC genotype in children with asthma compared to healthy children [239,240].

### 3.3. WISP1 in COPD

COPD is a progressive lung disease characterized by chronic airway inflammation, airway remodeling, airflow obstruction, and destruction of the alveolar parenchyma. The onset and advancement of COPD have been linked, in part, to persistent inflammation and ongoing degradation of the ECM, along with structural cell demise. These processes collectively result in the lung’s diminished capacity to trigger its own repair mechanisms [241].

In COPD, the activity of the WNT/β-catenin signaling pathway is generally reduced in lung epithelial cells, resulting in airway inflammation and the production of inflammatory cytokines via a PPARδ/p38 MAPK pathway by epithelial cells [25,26,148,242,243]. There are several mechanisms that contribute to this downregulation. Cigarette smoke is the predominant risk factor for COPD and plays a critical role in reducing WNT/β-catenin signaling. Cigarette smoke exposure decreased the expression of Frizzled receptor 4 (FZD4) in alveolar epithelial cells and increased the phosphorylation of β-catenin [244]. This phosphorylation event led to β-catenin degradation, thereby inhibiting the classical WNT signaling pathway. As a result, epithelial cell proliferation and alveolar repair processes were impaired, contributing to the progression of COPD. Moreover, in COPD patients, the expression of WNT-5A from fibroblasts was elevated. Glycosylation of WNT-5A negatively regulated WNT/β-catenin signaling by reducing β-catenin stability [25]. This reduction in β-catenin stability impaired wound healing and the trans-differentiation of alveolar type II (ATII) cells, which are crucial for alveolar repair and regeneration [26]. Therefore, reactivation of the canonical WNT/β-catenin signaling in COPD was suggested to be beneficial [25,245,246].

There is considerable information on the involvement of the canonical WNT/β-catenin pathway in COPD [43]. Oxidative stress, characterized by an imbalance between reactive oxygen species (ROS) production and the antioxidant defense system, is a key mechanism underlying lung injury and COPD development. Both exogenous (e.g., cigarette smoke) and endogenous sources contribute to increased ROS levels in the lungs. Several studies have linked oxidative stress to the WNT/β-catenin pathway. Reactive oxygen species, such as hydrogen peroxide (H2O2), have been shown to induce dephosphorylation and stabilization of β-catenin [247,248]. Additionally, mitochondrial ROS have been implicated in promoting the dissociation of Disheveled from its complex with nucleoredoxin, leading to an enhanced WNT/β-catenin signaling efficiency [249,250]. Another study demonstrated that the activation of the WNT-3A/β-catenin pathway selectively suppressed pro-inflammatory mediator expression and airway enlargement by upregulating the Nrf2 pathway [246].

However, so far, there has been no specific information on the involvement of WISP1 in COPD. The role of WNT/β-catenin signaling in COPD is complex, with evidence suggesting that both impaired and aberrant activation of this pathway contribute to disease pathology [251]. Both pathways require the binding of different WNT ligands, which may compete and influence each other’s activity [244]. For example, non-canonical WNT signaling, mediated by WNT-5A, can inhibit WNT-3A-induced canonical WNT/β-catenin signaling [252]. Notably, the WNT/β-catenin pathway was reported to be indeed activated in the airway epithelium in COPD as indicated by the upregulation of this pathway compared to non-smokers and control smokers [253]. This activation inhibited epithelial differentiation, polarity, and barrier function while induced EMT related to TGF-β [253].

### 3.4. MIF in COPD

MIF levels in COPD have been found elevated within lung tissue both in human and mouse studies [254,255]. Contrarily, other studies noted lower MIF levels in circulation in COPD patients [256,257], suggesting a localized protective response of MIF directly in the area affected by the disease [45]. Particularly, lower circulation MIF levels were associated with more severe COPD, while during acute exacerbations, circulating levels of MIF increased, indicating that exacerbation might trigger a systemic response involving MIF. Cigarette smoke also appears to elevate MIF levels in lung tissues, suggesting that elevated MIF levels may be a response to cigarette smoke-related injury. Another study, conducted in murine models, supported that MIF may contribute to emphysema pathogenesis by maintaining vascular homeostasis under oxidative stress [257]. Further, it was shown that the response to cigarette smoke was age-dependent, while MIF knock-out mice developed spontaneous emphysema and were more prone to cigarette smoke-induced emphysema [257]. After all, MIF is known to induce type 2 alveolar epithelial proliferation via CD74 [258]. Therefore, in the absence of MIF, the alveolar epithelium could not recover after injury [259]. Interestingly, MIF can have both protective and deleterious effects on COPD pathogenesis. Variations in the MIF gene may influence the extent to which COPD affects lung function [260], as evidenced by the MIF-794 CATT5 allele association with reduced DLCO (Diffused Capacity for Carbon Monoxide) [261,262]. Additionally, MIF levels vary depending on the GOLD stage, the type of components or particles to which patients were exposed, and whether patients experienced exacerbations [45].

## 4. WISP–MIF Axis: A Novel Regulatory Pathway in Immunity and Inflammation?

The ECM of the lung contains over 150 structural proteins within the basement membrane and interstitial matrix, interacting with multiple molecules, including enzymes, growth factors, and non-structural proteins [263,264]. Collagens, elastin, matrix metalloproteinases, and other matrix effectors support lung structure and functions with disease, leading to abnormal ECM deposition and impaired lung function [265,266]. The ECM also affects how the immune system responds, regulating inflammation and injury [267,268]. Additionally, it serves as a crucial bioactive component of the cellular microenvironment, influencing various cellular processes [269,270,271]. The matricellular protein WISP1 and MIF cytokine are key components of the ECM milieu, playing crucial roles in regulating immune responses. They are both significant in the context of lung diseases, including asthma and COPD (Table 2). These diseases involve complex interactions between structural cells, immune cells, and the ECM components.

WISP1 plays a key role in lung health and disease. It interacts with various components in the ECM, such as collagen and fibronectin, as well as with integrins that mediate cell–ECM interactions [61,104,272]. These interactions are important for cell functions such as adhesion, motility, and signaling, especially during inflammation and tissue injury in the lung [76,111,273,274]. During inflammation or injury, ECM remodeling occurs, involving changes in ECM composition and organization. In the context of VILI (ventilator-induced lung injury), the role of WISP1 in modulating TLR4-mediated inflammatory responses highlights its importance in how the ECM and cellular responses, including those of macrophages, are coordinated during injury and inflammation. WISP1 might act as an adapter or modulator of TLR4-mediated signaling, amplifying cytokine production through pathways such as NF-κB.

The role of WISP1 is not limited to the lung but is also relevant to other contexts, influencing both tissue injury responses and cancer progression. WISP1 plays a pivotal role in modulating immune responses across various pathologies by orchestrating the expression of chemokines and adhesion molecules [275,276]. For example, in melanoma, WISP1 knock-out leads to increased CD45(+) tumor-infiltrating leukocytes, including natural killer cells and CD8(+) T cells, yet impairs IFN-gamma release by CD8(+) T cells, affecting both immune cell presence and activity within the tumor microenvironment [277]. Additionally, WISP1 influences monocyte–macrophage trafficking and promotes M2 macrophage polarization, influencing systemic inflammation and tissue repair [101,278]. This is exemplified in bone graft resorption following intermittent hypoxic therapy, where WISP1 modulates cytokine levels such as IL-6, IL-17a, IL-17f, and IL-23r [279]. These functions highlight WISP1’s critical role in shaping immune responses. Furthermore, WISP1 is a pivotal regulator in tissue repair and fibrosis, with significant impacts on fibroblasts and stromal cells.

WISP1 has been shown to affect cell behavior in the lung by activating signaling pathways such as WNT/β-catenin and TGF-β, which regulate inflammation and repair processes [101,280]. In pulmonary fibrosis, WISP1 promotes fibrotic tissue remodeling by stimulating fibroblast proliferation and differentiation into myofibroblasts, key drivers of fibrosis progression. Similarly, in asthma, WISP1 can influence airway remodeling by affecting epithelial cell proliferation and ECM deposition [270], while in the context of mucosal wound repair, WISP1, which is produced by IL-10 from macrophages, promotes epithelial cell proliferation and wound closure by activating pro-proliferative pathways [280]. This activation directly impacts ECM remodeling during the wound-healing process.

Furthermore, WISP1 also regulates several key signaling pathways, such as PI3K, Akt, β-catenin, and mTOR. These pathways control immune cell behavior, cell death, and cell maintenance, impacting inflammation and oxidative stress. Therefore, WISP1 regulates immune cell behavior and recruitment in various diseases, including those involving immune system dysfunction [276]. Importantly, WISP1 links mechanical stress to immune responses by influencing immune cell recruitment and EMT in alveolar type II epithelial cells [103]. WISP1, which is a WNT target gene, is significantly upregulated in a hyaluronan- and MyD88-dependent manner, linking mechanical stress to immune signaling pathways and fibrosis [103]. Further, Akt phosphorylation and the subsequent activation of β-catenin in WISP1-stimulated cells suggest that WISP1 may involve autocrine feedback mechanisms, regulating its own expression in a β-catenin-dependent manner, as observed in glioblastoma stem cells [119]. These roles suggest that WISP1 is crucial in both lung repair and pathological conditions where tissue remodeling occurs.

Notably, WISP1 has also been recognized to be an important key player in other diseases, such as osteoarthritis (OA), further reinforcing the idea that WISP1 is a central mediator of pathological processes involving the ECM, making it an important target for therapeutic development [281,282]. In OA, WISP1 contributes to cartilage degradation by modulating chondrocyte activity and matrix integrity [283]. Mechanistically, WISP1 has been shown to influence the balance between anabolic and catabolic processes in articular cartilage, primarily through its interaction with key signaling molecules such as β-catenin and MMPs, especially MMP-13, which degrades collagen [284]. Elevated levels of WISP1 in OA cartilage are linked to the activation of the WNT/β-catenin pathway, which leads to an increased production of MMPs, thereby promoting ECM degradation [281,285]. Moreover, WISP1 promotes the expression of pro-inflammatory cytokines, such as IL-6 and TNF-α, which further exacerbate cartilage degradation and inflammation in the joint environment [286]. Furthermore, mechanical stress in OA upregulates WISP1, driving chondrocyte hypertrophy and apoptosis, which further accelerates extracellular matrix breakdown, making WISP1 a potential therapeutic target. However, there is also evidence suggesting that WISP1 can have protective effects in OA by preventing chondrocyte senescence and apoptosis through the activation of the αvβ3 receptor and the PI3K/Akt signaling pathway [287]. This indicates that WISP1’s role in OA is more complex than purely pathogenic, as it may also contribute to maintaining cartilage health under certain conditions. Additionally, WISP1 has been shown to interact with TNF-α in dermal fibroblasts, playing a role in modulating innate immune responses during wound healing and tissue repair via the α5β1 integrin and TNF-α signaling pathways [115].

On the other hand, MIF is a cytokine involved in the immune response, playing a crucial role in inflammation and the regulation of immune cell activity. MIF influences the immune response by promoting the production of other pro-inflammatory cytokines, and it can also affect cell proliferation and survival [45,288]. While it interacts with ECM components indirectly by modulating the immune response and inflammation, MIF itself is not a structural part of the ECM [45,289,290]. In the lungs, MIF has been extensively reported to be implicated in chronic lung diseases by perpetuating chronic inflammation, contributing to tissue damage and airway remodeling [45,260]. However, MIF’s functions extend to various pathologies. In chronic inflammatory diseases such as RA and OA, MIF drives inflammation by promoting fibroblast proliferation, increasing pro-inflammatory cytokines such as TNF-α and IL-6 through ERK1/2 and NF-κB signaling [291,292,293,294]. Furthermore, MIF regulates MMPs that contribute to tissue damage while influencing immune cell behavior and modulating apoptosis, thereby leading to sustained inflammation and tissue remodeling [293,294]. MIF also significantly impacts autoimmune diseases such as systemic lupus erythematosus (SLE) and inflammatory bowel disease (IBD) by exacerbating inflammation [295,296]. It interacts with the NLRP3 inflammasome, promoting the release of pro-inflammatory cytokines such as IL-1α, IL-1β, and IL-18 [178]. This interaction perpetuates chronic inflammation, ECM remodeling, and immune dysregulation, contributing to the pathology of these diseases. In cardiovascular diseases, including atherosclerosis and heart failure, MIF contributes to inflammation and tissue remodeling in the myocardium and vascular walls, affecting disease progression [297,298]. MIF influences ECM remodeling by modulating MMPs and TIMPs, reduces oxidative stress via TPOR activity, and inhibits apoptosis through S-nitros(yl)ation and JAB1/CSN5 interaction [299,300,301].

From the above, WISP1 and MIF, while functionally distinct, share overlapping roles in inflammation and ECM remodeling. Importantly, in a recent study, we have highlighted the interplay between WISP1 and MIF in lung diseases [104]. The ability of WISP1 to upregulate MIF through EGFR and Src kinases suggests a direct regulatory interaction. This interaction potentially amplifies the inflammatory and fibrotic responses seen in lung diseases such as asthma and COPD [104]. Given their roles in similar pathological processes and their impact on key signaling pathways, it is plausible that WISP1 and MIF operate within a coordinated axis. WISP1 influences immune responses and tissue balance through complex mechanisms, including its ability to regulate MIF. MIF, in turn, plays a key role in immune regulation and inflammation, driving further cytokine release and sustaining inflammatory responses.

## 5. Clinical Implications and Future Remarks

The interaction between WISP1 and MIF may introduce new dimensions to our knowledge of immune regulation. Understanding this axis could open up possibilities for targeted therapies in inflammatory diseases. Modulating WISP1 and/or MIF activity could provide new strategies to treat conditions such as autoimmune diseases, chronic inflammatory conditions such as asthma and COPD, and even cancer. However, research into the WISP1–MIF axis is still in its early stages, but the potential implications could be vast.

WISP1 plays a critical role in asthma pathogenesis by contributing to airway remodeling through the promotion of ASM hypertrophy and proliferation, influenced by the Akt signaling pathway and non-canonical TGF-β signaling [132]. It is also implicated in ECM deposition and is upregulated by TNF-α, highlighting its significance in the inflammatory and structural changes characteristic of asthma [220]. Therefore, WISP1 targeting would potentially mitigate airway remodeling, reduce inflammation, and improve lung function, making it a promising therapeutic strategy for managing asthma. In COPD, although WISP1’s involvement in lung function and ECM deposition indicates its role in structural changes seen, the complex role of WNT/β-catenin signaling, with both impaired and aberrant activation, necessitates precise therapeutic targeting to avoid exacerbating the disease [148]. As far as MIF is concerned, it has been reported to contribute to asthma pathogenesis and severity by promoting inflammation and cell proliferation, with genetic factors influencing its expression [45,232]. Interestingly, recent studies have demonstrated that MIF induces resistance to glucocorticoids in severe neutrophilic asthma, highlighting the potential of targeting MIF as a therapeutic strategy to enhance glucocorticoid responsiveness and alleviate inflammation in severe asthma [213]. Similarly, in COPD, MIF targeting might be a therapeutic option for managing inflammation and enhancing glucocorticoid responsiveness. However, MIF in COPD shows elevated levels in lung tissues, suggesting a protective response to cigarette smoke-induced injury, with lower circulating levels associated with more severe disease [188]. Furthermore, MIF’s dual role in different types of inflammation (Th2-related in asthma and possibly Th1 in other conditions) indicates that therapies must be carefully tailored to avoid unintended consequences [302].

Future studies will likely focus on detailed investigations into how WISP1 and MIF interact at the molecular level in immunity and inflammation. Developing drugs or biological agents that can specifically target the WISP1–MIF axis could offer new treatments for a range of inflammatory and immune-mediated conditions. Additionally, identifying biomarkers associated with the WISP1–MIF axis could improve disease diagnosis and monitoring, thereby advancing personalized treatment approaches. These areas of research hold promise for enhancing our understanding of the ECM–immunity interface and developing novel therapeutic strategies.

## Figures and Tables

**Table 1:** WISP1 affects lung homeostasis in various pulmonary diseases.

Disease	Roles of WISP1	References
Asthma	Induces airway remodeling, promotes pro-inflammatory factors, facilitates fibroblast migration, and causes hypertrophy and hyperplasia of airway smooth muscle cells	[76,132]
Pulmonary Fibrosis	Promotes fibroblast activation and myofibroblast differentiation, leading to excessive fibrosis	[61,104,106]
Lung Cancer	Enhances cell proliferation and survival and influences the tumor microenvironment	[145,146,147]
COPD	Contributes to inflammation and airway remodeling	[148,149,150]
Acute Lung Injury/ARDS	Exacerbates inflammatory responses and abnormal tissue repair	[85,113]

**Table 2:** Roles and common actions of WISP1 and MIF in asthma and COPD.

Action		WISP1	MIF	Common Actions	References
Role in Airway Remodeling	Asthma	Promotes ASM hypertrophy and proliferation via the PI3K/Akt/GSK-3β pathway, leading to collagen deposition and ECM remodeling. Stimulates fibroblast proliferation and ECM component production (e.g., Col1alpha1, FN1).	Promotes ASM proliferation and eosinophil migration via ERK1/2/Drp1 axis, leading to ECM deposition and airway remodeling. Enhances TGF-β1 accumulation and collagen deposition.	Both WISP1 and MIF enhance airway remodeling by promoting ASM proliferation and ECM deposition through distinct pathways: PI3K/Akt (WISP1) and ERK1/2 (MIF).	[76,216,225,231,234,235]
	COPD	Promotes ASM hypertrophy and ECM remodeling, exacerbated by oxidative stress and impaired epithelial repair, leading to chronic inflammation.	Promotes ASM proliferation and ECM deposition, exacerbated by oxidative stress and cigarette smoke exposure, contributing to chronic inflammation.		[45,254,255,257]
Interaction with TGF-β Signaling	Asthma	Upregulated by TGF-β through the PI3K/Akt/GSK-3β pathway, enhancing remodeling processes. Promotes sustained expression and remodeling effects through non-canonical TGF-β signaling.	Promotes TGF-β signaling by enhancing autophagy and collagen deposition via ERK1/2. MIF inhibition reduces TGF-β1 accumulation.	Both WISP1 and MIF modulate TGF-β signaling pathways, enhancing airway remodeling. WISP1 acts through PI3K/Akt, while MIF acts through ERK1/2.	[76,225]
	COPD	Upregulated by TGF-β through the PI3K/Akt/GSK-3β pathway, contributing to sustained remodeling and inflammation.	Enhances TGF-β signaling, leading to increased collagen deposition and inflammation. MIF inhibition reduces TGF-β1 levels.		
Impact on Inflammation	Asthma	Increased by TNF-α through WNT/β-catenin signaling, linking to chronic inflammation and remodeling in asthma.	Enhances inflammation by promoting ASMC proliferation and eosinophil migration via ERK1/2 and NF-κB pathways. Contributes to glucocorticoid resistance. Involved in Th2-related inflammation, promoting eosinophil migration. Also linked to glucocorticoid resistance in severe neutrophilic asthma by reducing annexin-A1 activity and promoting NLRP3 inflammasome activation.	Both WISP1 and MIF are involved in pro-inflammatory signaling, contributing to chronic inflammation.	[42,165,204,213,215,216,225,231,232,233]
	COPD	Increased by TNF-α and exacerbated by oxidative stress, contributing to chronic inflammation and impaired repair.	Increases inflammation by promoting ASMC proliferation and neutrophil accumulation. Contributes to glucocorticoid resistance and NLRP3 inflammasome activation.		
Effect on Glucocorticoid Resistance	Asthma	Not directly associated with glucocorticoid resistance; may indirectly influence steroid responsiveness through its effects on inflammation and remodeling.	Promotes glucocorticoid resistance in severe neutrophilic asthma by inhibiting annexin-A1, enhancing NLRP3 inflammasome activation, and reducing the effectiveness of glucocorticoids.	While WISP1 does not directly induce glucocorticoid resistance, MIF’s role suggests that targeting both could improve glucocorticoid responsiveness by reducing inflammation and remodeling.	[208,211,223,224,225,226]
	COPD	May indirectly influence glucocorticoid resistance through its effects on remodeling and inflammation.	Induces glucocorticoid resistance by inhibiting annexin-A1, increasing neutrophil accumulation and inflammation.		
Response to Environmental Factors	Asthma	Modulated by cigarette smoke and oxidative stress, impacting WNT signaling and epithelial repair.	Elevated by cigarette smoke exposure, contributing to inflammation and disease progression.	Both WISP1 and MIF respond to environmental factors such as cigarette smoke, exacerbating inflammation and remodeling.	[25,230,231,232,234,235,242,243,244,245]
	COPD	Modulated by environmental factors such as oxidative stress and cigarette smoke, affecting inflammation and repair.	Elevated in response to cigarette smoke, influencing inflammation and disease severity in COPD.		
